# Caregiving burden, social support, and psychological well-being among family caregivers of older Italians: a cross-sectional study

**DOI:** 10.3389/fpubh.2024.1474967

**Published:** 2024-10-23

**Authors:** Ramona Bongelli, Gianluca Busilacchi, Antonio Pacifico, Michele Fabiani, Carmela Guarascio, Federico Sofritti, Giovanni Lamura, Sara Santini

**Affiliations:** ^1^Department of Political Science, Communication and International Relations, University of Macerata, Macerata, Italy; ^2^Department of Economics and Law, University of Macerata, Macerata, Italy; ^3^INRCA IRCCS, Centre for Socio-Economic Research on Ageing, Ancona, Italy

**Keywords:** caregiving burden, social support, psychological well-being, older adults, Central Italy, long-term care (LTC)

## Abstract

**Objectives:**

This study aimed to identify factors affecting the psychological well-being of family caregivers of dependent older adults in Italy. Understanding these variables is essential for designing interventions to prevent negative outcomes in at-risk caregivers. The research explored how varying levels of caregiving burden and types of perceived social support influence psychological well-being.

**Methods:**

A cross-sectional study was conducted among 387 family caregivers of older adults in the Marche region (Italy) between November 2019 and March 2020. Data were collected via a structured questionnaire assessing psychological well-being (WHO-5 Well-Being Index), caregiving burden, and social support (COPE Index). Statistical analyses were performed using Jamovi software, with a significance threshold set at *p* < 0.05.

**Results:**

A significant negative correlation was found between caregiving burden and psychological well-being [*r* (364) = − 0.540, *p* < 0.001], with caregiving burden being a significant predictor of psychological well-being reduction (*R*^2^ = 0.290; *F* = 150, *p* < 0.001). A threshold value of 2 (on a 1–4 scale) was identified, where caregiving burden predicted a significant reduction in psychological well-being. Conversely, greater perceived social support was positively correlated with better psychological well-being [*r* (357) = 0.348, *p* < 0.001] and was a significant predictor of it [*R*^2^ = 0.121; *F* = 49.2, *p* < 0.001]. Support from social and health services had the most notable impact on psychological well-being. Moreover, social support mitigated the negative impact of caregiving burden on psychological well-being.

**Conclusion:**

The study confirms that high caregiving burden adversely affects caregivers’ psychological well-being, while social support plays a protective role. These findings highlight the need for interventions focused on reducing caregiving burden and enhancing support systems for family caregivers.

## 1 Introduction

The anticipated increase in the older adult population in many countries worldwide, including Italy, represents a significant challenge. According to demographic projections by the Istituto Nazionale di Statistica (ISTAT),^1^ by 2050, individuals aged 65 and over are expected to represent 34.9% of the Italian population. This demographic shift is likely to place considerable pressure on social protection systems and significantly increase the demand for older adult assistance services, as well as the involvement of family members in their care. Currently, much care is provided informally by family members (1), who play a crucial role in supporting frail older adults – those who “are infirm and experience significant difficulties in performing activities of daily living, resulting in a lack of independence and the need for extensive assistive care,” as defined by the APA dictionary.^2^ This type of care, also known as long-term care (LTC), encompasses a wide range of personal, social and medical services aimed at assisting individuals (particularly adults aged 60 years or above with functional or cognitive impairments) who are unable to perform activities such as eating, dressing and bathing independently.^3^

With the expected progressive growth of the older adult population, it is highly likely that the responsibilities of family caregivers will continue to intensify, exposing them to increasing strain and stress, thereby raising the urgent question of how to support them.

As previously mentioned, family caregivers are often responsible for the daily care of frail older adult relatives. While these care tasks are essential, they often come at a high personal cost. Numerous studies have demonstrated the negative impact of caregiving burden on the quality of life, health status and psychological well-being of family caregivers [e.g., (2, 3, 4–10, 54)]. In the Italian context, research has shown that family caregivers of frail older adults face significant challenges in maintaining their psychological well-being while managing the increasing demands of caregiving [e.g., (1, 11)].

Although there is considerable agreement about the detrimental effects of caregiving, much research has also highlighted the positive experiences associated with caring, such as a sense of fulfillment and satisfaction [e.g., (12–17, 53)]. However, these positive aspects often fail to compensate for caregiving fatigue, especially when care responsibilities are prolonged over time. Indeed, in many cases, the multiple demands of caregiving lead to burnout (18), stress (19), and other negative psychological outcomes [e.g., (20–22)].

A key factor that has been shown to mitigate the effects of caregiving burden is perceived social support, both emotional and instrumental. It has been consistently associated with lower levels of subjective distress and improved psychological well-being [e.g., (23–25)]. In the Italian context, research has also indicated that social support can play a crucial role in buffering the negative effects of caregiving [e.g., (26)], but the specific types of support that caregivers perceive as most beneficial and the extent to which they can effectively mitigate caregiving stress remain under-explored aspects.

Thus, despite the valuable insights provided by previous studies, several gaps remain. Firstly, there is a lack of knowledge about when caregiving becomes excessively burdensome and starts to negatively impact caregivers’ psychological well-being. In other words, while much of the research focuses on the general effects of caregiving, few studies have identified the specific threshold at which the burden seems to become detrimental. Secondly, although the protective role of social support is widely recognized, there is a limited understanding of which types of support caregivers perceive as most effective in reducing the negative impacts of caring. Furthermore, the complex interaction between caregiving burden, social support and psychological well-being has not been adequately examined in a comprehensive manner, especially in the Italian context.

This study aims to fill the aforementioned gaps by (1) investigating the extent to which caregiving burden negatively impacts psychological well-being among family caregivers, (2) identifying the specific aspects of social support that most affect caregivers’ psychological well-being, and (3) examining whether and how social support can mediate the relationship between caregiving burden and psychological well-being. In other words, the primary aims of the present research are (1) to determine how caregiving burden affects psychological well-being among Italian family caregivers of older adults; (2) to explore the role of specific aspects of social support in affecting family caregivers’ psychological well-being; and (3) to identify whether social support acts as a buffer to alleviate the psychological strain due to caregiving.

These objectives will therefore be pursued by answering the following research questions (RQs):

- *RQ1*: Does caregiver burden affect psychological well-being? If so, to what extent?
- *RQ2*: Does social support influence psychological well-being? If so, which aspects have the most significant impact on caregivers’ psychological well-being?
- *RQ3*: If caregiver strain negatively impacts caregivers’ psychological well-being, can social support mediate this effect (i.e., act as a buffer)?

The answers to these questions could provide useful indications for the development of interventions aimed at reducing the burden on caregivers, enhancing their psychological well-being and improving the care they provide.

## 2 Materials and methods

### 2.1 Data collection and sampling

This cross-sectional study draws on data collected in the Marche region (Italy) by the survey titled ‘*The perspective of older people with LTC needs and their family caregivers in the Marche region’* (27), commissioned by the Marche Region Authority to deepen the understanding of the care needs of vulnerable older people living in this area of Central Italy and their primary family caregivers.

The sampling followed a convenience/purposive method (28). Older people were identified first by the pensioners’ trade unions to which older individuals or their relatives had sought support for accessing LTC services, e.g., the State Care Allowance (“Indennità di Accompagnamento” in Italian), a monetary cash benefit provided by the Government to individuals with a severe level of disability.

Older individuals were included and considered eligible to participate in the study if they (a) signed the informed consent form and volunteered to participate in the study, and (b1) received the State Care Allowance (which ensured a high degree of disability) or (b2) although they did not receive the State Care Allowance, they reported a score of less than 9 on a 12-item scale measuring their level of autonomy in Activities of Daily Living (ADL), based on the Barthel Index (29), and the Instrumental Activities of Daily Living (IADL (30)). This screening was conducted through face-to-face interviews administered by trained volunteers from the trade unions.

Once recruited, older individuals were screened for eligibility, and the interviewers planned an appointment to meet them in person and administer the questionnaire at their homes. The interviewers then asked them to indicate their primary family caregiver to also participate in the survey. Subsequently, the interviewer contacted the primary caregiver, screened them and if eligible, scheduled another appointment at the caregiver’s home or in the trade union office.

Family caregivers – who were all Italian-speaking – were included in the sample if they were caregivers of older individuals with LTC needs, were 25 years of age or older, had been providing direct assistance to the older relative for at least 1 year, did not have a cognitive impairment and signed the informed consent.

For older individuals, we did not conduct a sample size study; however, to ensure the highest correspondence between the sample selected for this study and the universe of older people with LTC needs living in the Marche region, we estimated the share of the over-75 population with severe mental and physical limitations for each of the 13 health districts in which the health service of the Marche Region is articulated (at this level, care pathways are activated and integration between health and social activities is realized). This share was then stratified by age (using three groups: 75–79, 80–84, and 85+) and gender, and the results were used as a basis to identify the minimum number of respondents to be interviewed in each district (31). The interviewers visited the interviewees’ homes and administered the questionnaire face-to-face. In about half of the cases, the person in need of care was unable to fill in the survey unassisted, and the primary caregiver completed the questionnaire as a proxy for them for all questions not requiring a subjective response.

As for the caregivers, the sample size was determined based on the older individuals’ sample size.

All data were collected anonymously, in compliance with the guidelines set forth in EU Regulation No. 679 of the European Parliament and of the Council of 27 April 2016, as well as the Helsinki Declaration (2013). The study was submitted for approval to the Ethics Committee of the National Institute of Health and Science on Aging (INRCA), which deemed approval unnecessary since the investigation did not involve clinical patients.

Data collection was carried out by the primary pensioners’ trade union volunteers between November 2019 and March 2020 in the aforementioned 13 health districts of the Marche Region, including both inland and coastal areas.

### 2.2 Measures

Considering the two targets, the survey comprised two common assessment tools: the first included a series of questions designed to assess the condition and requirements of frail older individuals with LTC needs; the second consisted of a set of questions specifically targeted at caregivers, aiming to gain insights into their condition and needs. Except for questions about ADL and IADL (29, 30), the questionnaire for older individuals did not include other psychometrically validated scales. The questionnaire for family caregivers embedded *ad-hoc* multiple-choice and open-ended questions on socio-demographic aspects, family caregivers’ burden, well-being, formal and informal support received, and work-life balance issues. It also included some validated scales for measuring caregiving burden (the 7-item subscale of the Carers of Older People in Europe Index - COPE Index), psychological well-being (the World Health Organization’s Five-Well-Being Index -WHO-5), and social support (the 4-item subscale of the Carers of Older People in Europe Index - COPE Index), which are described in detail below.

In this study, we focused exclusively on the outcome measures of the family caregivers’ questionnaire.

#### 2.2.1 Personal information data

Family caregivers answered a series of questions designed to identify the characteristics of the sample considered (e.g., gender, age, level of education, parental status with the older adult care recipient).

#### 2.2.2 Caregiver burden

Caregiver burden was measured by four specific questions tailored for this study, aiming to assess the extent to which caregivers perceive a loss of personal time due to caring for the older adult person (1. *Do you feel that you do not have enough time for yourself because of the time spent on the older adult person?*), as well as their perceived levels of stress resulting from caring for the older adult and coping with other responsibilities (2. *Do you feel stressed between caring for the older adult person and trying to cope with other responsibilities?*), levels of fatigue (3. *Do you feel fatigued when caring for the older adult person?*) and sense of the insecurity (4. *Do you feel insecure about what to do for your older adult person?*). Respondents were asked to rate on a 5-point Likert scale from 0 (never) to 4 (almost always). The internal consistency of the items was good, with a Cronbach’s alpha of 0.80. Additionally, caregiving burden was also measured by the 7-item subscale of the Carers of Older People in Europe Index (COPE Index) (2, 55, 56), specifically item 1. *Do you find caring too demanding?*; 2. *Does assisting create any difficulties for you in your relations with your friends?*; 3. *Does caring have a negative effect on your physical health?*; 4. *Does caring create difficulties for you in your relationships with your family?*; 5. *Does caring cause you financial difficulties?*; 6. *Do you feel ‘trapped’ in your role as a carer?*; 7. *Does caring have a negative effect on your emotional balance?* Respondents rated items on a 4-point Likert scale from 1 (always) to 4 (never), except for item 7, where the rating was reversed (1 = never, 4 = always). The values of the first six items were inverted to match item 7 and previous items. The scale showed good internal consistency (Cronbach’s alpha = 0.84). Following Balducci et al. (2), an exploratory factor analysis (EFA) – the results of which are not reported in detail due to space limitations – revealed a 3-factor structure, one of which is represented by the caregiver burden subscale, replicating Balducci et al.’s findings. To assess the construct validity of the COPE Index subscale, a confirmatory factor analysis (CFA) was also conducted on the seven items. All items loaded on the same factor, with estimates ranging from 0.480 to 0.723, supporting the unidimensional structure of the COPE Index Caregiver Burden subscale. The overall fit indices suggest that the model fits the data well, with a CFI of 0.973, a TLI of 0.959 (both values above 0.95) and a RMSEA of 0.0664 (i.e., below 0.08).

Furthermore, to account for the multiple components of care burden, we computed a new summary index called ‘total care burden’. Principal component analysis (PCA) and exploratory factor analysis (EFA) showed that all items (i.e., the four specifically tailored for this study and the 7 of the COPE subscale) loaded on a single factor, explaining 47.9% of the variance. The new index had high internal consistency, with a Cronbach’s alpha of 0.888.

#### 2.2.3 Well-being

The family caregivers’ psychological well-being was assessed using the Italian version of the World Health Organization’s Five-Well-Being Index (32–34), which is available in PDF format on the WHO-Five website at the following link.^4^ The WHO-5 is a self-report measure assessing subjective psychological well-being, consisting of five statements that respondents rate on a 6-point Likert scale ranging from 0 (not at all) to 5 (all the time). Respondents were asked to provide answers that come closest to how they have felt in the past 2 weeks to the following five assertions: *I have felt cheerful and in good spirits*; *I have felt calm and relaxed*; *I have felt active and vigorous*; *I woke up feeling fresh and rested*; *My daily life has been filled with things that interest me.* Higher scores correspond to a higher level of well-being; conversely, lower scores correspond to a lower level of well-being. The raw score is calculated by adding up the scores given by respondents for the five answers and ranges from 0 (lowest well-being) to 25 (highest well-being). A score below 13– as suggested by many studies in the literature [e.g., (35, 36, 57)] – indicates a poor state of well-being and is an indication to perform the depression test. The raw score can be multiplied by 4 to give a percentage score ranging from 0 (worst) to 100 (best). In this case, the cut-off is set at 50. Cronbach’s alpha and McDonald’s omega were both 0.90. To assess the construct validity of the WHO-5 Index, a CFA was conducted. The factorial loadings are strong (ranging from 0.694 to 0.866) and statistically significant, showing that each item contributes significantly to the latent factor. However, the Chi-square results [*χ*^2^ (5) = 54.9, *p* < 0.001] and the high RMSEA (0.162) indicate a misfit of the model to the data, despite the high values of CFI (= 0.96) and TLI (= 0.92). In other words, although CFI and TLI suggest that the model satisfactorily explains an important part of the variance, the high RMSEA deserves attention.

Respondents’ well-being was also measured by:

- The initial item of the SF-36 Health Survey (37–41), which assesses individuals’ overall perception of their *general health* on a 5-point Likert scale ranging from 1 (excellent) to 5 (poor). Nonetheless, we preferred to reverse values, so that 1 stands for poor and 5 for excellent;
- One question specifically tailored for this study to explore the caregiver’s assessment of their *quality of life* over the past 2 weeks. Again, respondents were asked to answer on a 5-point Likert scale ranging from 1 (very good) to 5 (very bad). In this scale, we preferred to reverse values, so that 1 would refer to very poor and 5 to very good.

Since our questionnaire measured well-being using different items that assess specific components, we calculated a new summary index called ‘total well-being’. PCA results indicated that all items loaded optimally on a single factor, explaining 63.6% of the variance. Similarly, EFA confirmed that all items loaded optimally on a single factor. Cronbach’s alpha for this new index was 0.897, indicating high internal consistency.

#### 2.2.4 Social support

Social support, a complex and multidimensional construct [see (42)] – encompassing both the general perception of being supported by friends and family networks, as well as by health and social services – was assessed through the 4-item subscale of the Carers of Older People in Europe Index (COPE Index) (2, 55, 56), specifically item 1. *Do you feel adequately supported by your friends/neighbors?*; 2. *Do you feel adequately supported by your family?*; 3. *Do you feel adequately supported by health and social services (public, private, or voluntary)?*; 4. *Overall, do you feel adequately supported in your role as an assistant?*). Respondents rated on a 4-point Likert scale from 1 (Never) to 4 (Always). This subscale measures the extent to which caregivers feel supported by their family, friends, and health and social services. In our sample, Cronbach’s alpha and McDonald’s omega were, respectively, 0.576 and 0.611. Although both values are poor, they are nevertheless acceptable (43–45). Specifically, since Cronbach’s alpha tends to increase with the number of items on the scale, adding more well-aligned items (in this case, for example, concerning other specific types of social support) could improve internal consistency. As the scale is multidimensional, refinement of the subscales could lead to better consistency in each dimension. A CFA was also conducted for this subscale. The factor loadings are all significant, but there is variability in the strength of their associations (which range from 0.317 to 0.736) with the latent factor. The fit indices suggest that the model has a moderate fit to the data. While the CFI is good (= 0.962), the RMSEA (= 0.0910, i.e., above 0.08), the TLI (= 0.887, i.e., below 0.90), and the Chi-square results [*χ*^2^ (2) = 8.41, *p* = 0.015] indicate that the model might require some improvements to better represent the underlying data.

Social support was also measured by two questions specifically tailored for this study to find out whether caregivers think they can rely on others, i.e., whether they can find someone to substitute in case of illness or need for a break. Respondents were asked to choose among three different possible answers: I could find quite easily (1); I could find with difficulty (2); I could not find anyone (3).

### 2.3 Data analysis

Data were analyzed using Jamovi (Version 2.3.21.0), an open statistical software,^5^ built on top of the R statistical language^6^, and RStudio. Both descriptive and inferential (*t*-test, ANOVA, correlations, linear regressions, Receiver Operating Characteristic – ROC - analysis, multivariate logistic regression, and mediation) analyses were performed. Descriptive statistics have been used to summarize the characteristics of the sample, including measures of central tendency (e.g., mean) and variability (e.g., standard deviation) for continuous variables. For categorical variables, frequencies and percentages were reported. Inferential statistics were used to show the relationships between continuous variables (e.g., caregiver burden and well-being), with Pearson’s correlation coefficients computed to assess the strength and direction of linear relationships. Additionally, linear regression models were performed to determine whether one variable predicts another. For categorical variables, tests such as Chi-square were used to examine the association between different groups. Missing data were handled systematically. Since the proportion of missing data was low, a complete-case analysis was performed. A standard level of statistical significance (*p*-value) was set at 0.05. Any p-value below this threshold was considered statistically significant. Alongside *p*-values, 95% confidence intervals (CIs) were reported for effect sizes in regression models and other inferential tests. CIs were calculated to provide a range of values within which the true parameter value likely lies. Reporting confidence intervals offers insight into the precision of the estimates and is more informative than p-values alone.

## 3 Results

### 3.1 Sample characteristics

Out of the 387 individuals who completed the questionnaire, 251 (64.9%) were female, while 136 (35.1%) were male. The participants’ ages ranged from 25 to 89, with a mean age of 63.3 (SD = 11.4). This value is particularly noteworthy as it indicates that our sample of family caregivers for dependent older adult individuals consists largely of individuals who are themselves older adult or nearing that stage. Moreover, over half (56.6%) of the family caregivers in our sample provide care without assistance from non-family caregivers, thus exclusively taking on the burden of caring. Among the participants, 62.5% were daughters or sons of the assisted older persons, while 21.4% were spouses. Most caregivers (55.6%) live in the same household as the person they are caring for, which could make caring even more stressful. On average, they spend 60.8 h per week caring for the older adult. For a more comprehensive overview of the sample characteristics, please refer to Supplementary Table S1.

In the following section, descriptive and inferential analyses concerning the variables taken into consideration will be presented.

### 3.2 Descriptive and inferential analyses

#### 3.2.1 Caregivers’ care burden

As mentioned in the methodology section, caregiver burden was first measured using four questions specifically designed for this purpose. Higher scores (4 = ‘almost always’) indicate a higher perceived care burden. As shown in Table 1, caregivers assigned an average score between 2 and 3 (indicating responses between ‘sometimes’ and ‘often’) to questions about lack of personal time, stress, and caregiving fatigue. They assigned an average score between 1 and 2 (indicating responses between ‘rarely’ and ‘sometimes’) to the question about insecurity related to caring for the older relatives. When combining the percentages of respondents who answered sometimes (2), often (3) and almost always (4) for the items on lack of personal time (74.4%), perceived stress (75.6%), and caregiving fatigue (70.4%), the overall rate for each item is approximately 70%. Only the item related to insecurity in providing care shows a distinct pattern, with a higher percentage of respondents assigning lower values, thus implicitly suggesting that they feel confident about caring for the person most of the time.

**Table 1:** Descriptive statistics and response frequencies for care burden items.

Care burden	*N*	Mean	Lower 95%CI	Upper 95%CI	SD	Variance	Never (0)		Rarely (1)		Sometimes (2)		Often (3)		Almost always (4)	
							*N*	%	*N*	%	*N*	%	*N*	%	*N*	%
1. Not having time for oneself	386	2.19	2.08	2.30	1.13	1.28	33	8.5	66	17.1	133	34.5	103	26.7	51	13.2
2. Feeling stressed	386	2.25	2.13	2.37	1.19	1.43	43	11.1	51	13.2	119	30.8	114	29.5	59	15.3
3. Feeling fatigue	386	2.07	1.95	2.19	1.21	1.47	54	14	60	15.5	125	32.4	99	25.6	48	12.4
4. Feeling of insecurity	386	1.38	1.26	1.49	1.15	1.33	105	27.2	115	29.8	101	26.2	45	11.7	20	5.2

The CI of the mean assumes sample means follow a t-distribution with N - 1 degrees of freedom.

To test whether gender and age affect the level of burden experienced, t-test and ANOVA were used (see Table 2). T-test (applied to the mean value of the four items) revealed no significant differences based on caregivers’ gender [*t* (384) = −1.35, *p* = 0.178]. However, when the four items were analyzed separately, a statistically significant difference emerged in terms of perceived stress for caring tasks and managing responsibilities, with women caregivers reporting significantly more stress than men [*t* (384) = −2.08, *p* = 0.038]. ANOVA test revealed instead that age significantly impacts the perceived burden of care [*F* (4, 128) = 3.67, *p* = 0.007]. Specifically, post-hoc tests indicated that caregivers over 80 perceive a significantly higher care burden than the 60–69 age group (*p* = 0.002).

**Table 2:** *T*-test and ANOVA values concerning the 4 custom-designed questions measuring care burden.

	Statistic test	Statistical value
Gender		
Care burden total score (mean score for the four items)	*T*-test	*t* = −1.35, df = 384, *p* = 0.178
1. Not having time for oneself	*T*-test	*t* = −1.18, df = 384, *p* = 0.237
2. Feeling stressed	*T*-test	*t* = −2.08, df = 384, *p* = 0.038*
3. Feeling fatigue	*T*-test	*t* = −1.90, df = 384, *p* = 0.059
4. Feeling of insecurity	*T*-test	*t* = −1.01, df = 384, *p* = 0.311
Age		
Care burden total score	ANOVA	*F* = 3.67, df1 = 4, df2 = 128, *p* = 0.007*

However, correlation analysis (Pearson’s r = 0.094, *p* = 0.066) and linear regression (*R*^2^ = 0.00880; *F* = 3.41, *p* = 0.06) showed no significant relationship between age (as a continuous variable) and perceived care burden, meaning that advancing age alone is not a reliable predictor of increasing perceived care burden.

In addition to the four items, caregiver burden was also measured using the 7-item subscale of the COPE index. As shown in Table 3, the respondents in our sample gave an average score between 2 and 3 (indicating answers between ‘sometimes’ and ‘often’) to items 1, 2, 6, and 7. Specifically, caregivers reported that they sometimes or often perceived their caregiving activities as being too demanding (mean = 2.53), responsible for difficulties with friends (mean = 2.08), a source of their feeling of being trapped in their role (mean = 2.10), and having negative effects on their own emotional well-being (mean = 2.04). Conversely, they assigned an average score between 1 (‘never’) and 2 (‘sometimes’) to items 3, 4, and 5, indicating that they only rarely or sometimes perceived their caregiving activities as being responsible for negative effects on their physical health (mean = 1.96), causing difficulties with their family (mean = 1.61), or leading to financial difficulties (mean = 1.72).

**Table 3:** Descriptive statistics for COPE index subscale measuring care burden.

Cope index subscale for care burden	*N*	Missing	Mean	Lower 95% CI	Upper 95% CI	SD	Variance
1. Do you find caregiving too demanding?	385	2	2.53	2.44	2.62	0.893	0.797
2. Does caregiving cause difficulties in your relationship with your friends?	385	2	2.08	1.98	2.18	0.986	0.972
3. Does caregiving have a negative effect on your physical health?	386	1	1.96	1.87	2.04	0.871	0.759
4. Does caregiving cause difficulties in your relationship with your family?	387	0	1.61	1.53	1.69	0.778	0.606
5. Does caregiving cause you financial difficulties?	385	2	1.72	1.63	1.81	0.907	0.823
6. Do you feel trapped in your role as caregiver?	386	1	2.10	2.01	2.20	0.975	0.950
7. Does caregiving have a negative effect on your emotional well-being?	386	1	2.04	1.95	2.13	0.901	0.811

The CI of the mean assumes sample means follow a t-distribution with N - 1 degrees of freedom.

Again, the t-test – calculated on the mean of the 7-item subscale of the COPE Index – showed no significant differences based on the gender of the caregiver [*t* (376) = −1.78, *p* = 0.076]. However, when the t-test was calculated on the seven items separately, there were statistically significant differences between men and women on the items measuring the negative impact of caregiving on physical health [*t* (384) = −2.34, *p* = 0.019] and emotional well-being [*t* (384) = −2.53, *p* = 0.012], with female caregivers reporting significantly more negative impacts of caregiving, both physically and emotionally. Regarding age, ANOVA test did not reveal any statistically significant differences across the various caregiver age groups [*F* (4, 123) = 1.56, *p* = 0.188] (see Table 4). It should be noted, however, that the mean scores of caregivers aged 70–79 and 80 and over were higher than 2, indicating that they often feel that caregiving has a negative impact on their relationships, physical health and emotional well-being. In contrast, younger age groups reported consistently lower mean scores.

**Table 4:** *T*-test and ANOVA values concerning Cope Index subscale measuring care burden.

	Statistical test	Statistic value
Gender		
Overall Cope Index subscale for care burden	*T*-test	*t* = −1.78, df = 376, *p* = 0.076
1. Do you find caregiving too demanding?	*T*-test	*t* = −0.7238, df = 383, *p* = 0.470
2. Does caregiving cause difficulties in your relationship with your friends?	*T*-test	*t* = −1.2232, df = 383, *p* = 0.222
3. Does caregiving have a negative effect on your physical health?	*T*-test	*t* = −2.3478, df = 384, *p* = 0.019*
4. Does caregiving cause difficulties in your relationship with your family?	*T*-test	*t* = −1.6848, df = 385, *p* = 0.093
5. Does caregiving cause you financial difficulties?	*T*-test	*t* = 0.0591, df = 383, *p* = 0.953
6. Do you feel trapped in your role as caregiver?	*T*-test	*t* = −0.6655, df = 384, *p* = 0.506
7. Does caregiving have a negative effect on your emotional well-being?	*T*-test	*t* = −2.5350, df = 384, *p* = 0.012*
Age		
Cope Index subscale for care burden	ANOVA	*F* = 1.56, df1 = 4, df2 = 123, *p* = 0.188

Even when age is treated as a continuous variable, correlation analysis (Pearson’s r = 0.093, *p* = 0.072) and linear regression analysis (*R*^2^ = 0.00857, *F* = 3.25, *p* = 0.072) do not reveal significant relations, again confirming that the age of the caregiver alone is not a predictor of increased perceived care burden.

Although the four items specifically designed in this study to measure care burden and the COPE index subscale measure different aspects of perceived caregiving burden, they appear to be complementary features of the same dimension. This is indicated by the results of the correlation analysis (which was carried out after converting all scores into z-scores to standardize the measures), according to which Pearson’s r coefficient was found to be 0.691, a highly significant value (*p* < 0.001), and it is also confirmed, as previously mentioned in the methodological section, by PCA and EFA that showed that all items load optimally on a single factor, we labeled ‘total care burden’.

#### 3.2.2 Caregivers’ well-being

In terms of psychological well-being, as measured by the WHO-5 scale, descriptive analysis reveals that more than half of the caregivers in our sample scored below the cut-off point of 13, which suggests the administration of a depression test. The mean score was 11.1 (SD ± 6.27). Although a clear trend emerged, with women showing lower well-being scores than men, this difference is not statistically significant [*t* (372) = 1.89, *p* = 0.059]. Conversely, when data are split by age, ANOVA indicates a clear pattern, according to which psychological well-being significantly declines with increasing age [*F* (4, 134) = 3.31, *p* = 0.005] (see Table 5).

**Table 5:** WHO-5 scores and statistical tests.

Group	Total (N)	WHO-5 raw score	SD	Below the threshold		Above the threshold		Total (%)	Statistic test	Statistical value
				*N*	%	*N*	%			
Gender									T-test (gender)	*t* = 1.89, df = 372, *p* = 0.059
M	132	11.09	6.27	63	16.8% (30.1%)	69	18.4% (41.8%)	35.3% (100%)		
F	242	10.06	6.23	146	39.0% (69.9%)	96	25.7% (58.2%)	64.7% (100%)		
Tot.	374	11.01	6.27	209	55.9% (100%)	165	44.1% (100%)	100%		
Age									ANOVA (age)	*F* = 3.31, df1 = 4, df2 = 134, *p* = 0.005*
70–79	57	10.07	6.28	34	9.1% (16.3%)	23	6.1% (13.9%)	15.2% (100%)		
60–69	139	11.42	6.27	70	18.7% (33.5%)	69	18.4% (41.8%)	37.1% (100%)		
50–59	101	11.18	5.98	58	15.5% (27.8%)	43	11.5% (26.1%)	27.0% (100%)		
≥ 80	42	8.74	5.93	34	9.1%, (16.3%)	8	2.1% (4.8%)	11.2% (100%)		
Under 50	35	14.06	6.36	13	3.5% (6.2%)	22	5.9% (13.3%)	9.4% (100%)		
Tot.	374	11.01	6.27	209	55.9% (100%)	165	44.1% (100%)	100%		

As revealed by the post-hoc test, the significance is due to the difference between the younger (under 50 years) and the older group (70–79 years, *p* = 0.023; and 80 and over, *p* = 0.002).

These results are also confirmed by correlation (Pearson’s r coefficient was found to be −0.181, *p* < 0.001), and linear regression analyses (*R*^2^ = 0.0328; *F* = 12.6, *p* < 0.001), which suggest that increasing age of the caregiver is a predictor of lower psychological well-being.

Since well-being is a complex construct, in addition to measuring it using the WHO-5 scale, it was also assessed using two other items: one measuring the overall perception of general health and the other quality of life.

As for the *general health status*, family caregivers’ overall perception was rated by most of the respondents (46.5%) as *fair*. The average rating was 2.59 on a scale ranging from 1 (poor) to 5 (excellent). Although some differences in the well-being of caregivers based on gender were found, with men reporting higher levels of well-being (average score of 2.70) compared to women (average score of 2.53), these differences were not statistically significant, as indicated by the t-test results [*t* (379) = 1.91, *p* = 0.056]. Conversely, ANOVA test showed statistically significant differences according to age [*F* (4, 129) = 9.96, *p* < 0.001] (see Table 6).

**Table 6:** Caregivers’ perceived health status: frequencies, percentages and statistical tests.

	Frequencies	%	M	SD	Statistic test	Statistical value
Health status overall perception score						
1. Poor	16	4.2%				
2. Fair	177	**46.5%**				
3. Good	142	37.3%				
4. Very good	39	10.2%				
5. Excellent	7	1.8%				
Tot.	381	100%				
Health status score by gender						
					T-test	*t* = 1.91, df = 379, *p* = 0.056
Male	135		2.70	0.858		
Female	246		2.53	0.765		
Tot.	381		2.59	0.802		
Health status score by age						
					ANOVA	(*F* = 9.96, df1 = 4, df2 = 129), *p* < 0.001*
70–79	59		2.31	0.701		
60–69	143		2.57	0.774		
50–59	102		2.73	0.869		
≥ 80	42		2.31	0.680		
Under 50	35		**3.11**	0.676		

Specifically, the post-hoc test revealed that younger caregivers, i.e., those aged under 50, rate their health as significantly better (mean value = 3.11) than older caregivers, particularly those aged 60–69, 70–79, and 80-plus. However, there is no significant difference in health ratings between younger caregivers and those aged 50–59. Conversely, the over-80s rate their health as significantly worse (mean value = 2.31) than those aged 50–59. Finally, the group aged 50–59 rated their health as significantly better (mean value = 2.73) than the group aged 70–79 (mean value = 2.31). These results are statistically confirmed by correlation (Pearson’s r coefficient was found to be −0.298, *p* < 0.001) and linear regression (*R*^2^ = 0.0890; *F* = 37.0, *p* < 0.001) analyses, which indicate that caregiver’s general health status perception worsens as age increases. In other words, increasing age of the caregiver is a predictor of worsening health.

As for the *perceived quality of life over the past 2 weeks*, a high percentage of family caregivers (46.7%) assessed it as ‘Neither good nor poor’. The average rating on a scale of 1 (very poor) to 5 (very good) was 3.38. Since higher scores indicate higher perceived quality of life, the caregivers in our sample seem to be near the middle of the scale. Although there are some gender-related differences in quality of life, with men assigning higher mean values (3.46) compared to women (3.34), this difference is not statistically significant [*t* (381) = 1.63, *p* = 0.105]. Conversely, ANOVA test showed statistically significant differences based on age [*F* (4, 129) = 7.04, *p* < 0.001]. Like the results for the general health assessment, caregivers’ ratings of their own quality of life are significantly influenced by age (see Table 7). The post-hoc test shows statistically significant differences, with younger caregivers (under 50) reporting better quality of life scores compared to older caregivers. Specifically, those over 80 rated their quality of life significantly lower than younger groups, especially those aged 50–59 and 60–69. These findings are further supported by the correlation analysis (Pearson’s r = −0.205, *p* < 0.001) and linear regression analysis (*R*^2^ = 0.0393; *F* = 16.6, *p* < 0.001), both of which indicate that as caregivers age, their quality of life decreases. In other words, advancing caregiver age is a predictor of lower quality of life.

**Table 7:** Caregivers’ perceived quality of life: frequencies, percentages and statistical tests.

	Frequencies	%	*M*	SD	Statistic test	Statistical value
Quality of life overall perception score						
1.Very poor	2	0.5%				
2.Poor	31	8.1%				
3.Neither good nor poor	179	**46.7%**				
4.Good	161	42.0%				
5.Very good	10	2.6%				
Tot.	383	100%				
Quality of life score by gender						
					*T*-test	*t* = 1.63, df 381, *p* = 0.105
Male	135		3.46	0.667		
Female	248		3.34	0.707		
Tot.	383		3.38	0.695		
Quality of life score by age						
					ANOVA	*F* = 7.04, df1 = 4, df 2 = 129. *p* < 0.001*
70–79	59		3.31	0.650		
60–69	144		3.40	0.713		
50–59	101		3.40	0.634		
≥ 80	43		3.02	0.707		
Under 50	36		3.81	0.624		

Since our questionnaire assessed well-being through several components (psychological well-being, general health, perceived quality of life), we conducted – after transforming all the scores of the different variables into z-scores to standardize the measures – a correlation analysis. Its results reveal that caregivers’ subjective assessment of their own health status correlates positively and significantly with their assessment of their own quality of life [*r* (376) = 0.39, *p* < 0.001], thus, a better assessment of one’s health corresponds to a better assessment of one’s quality of life. Similarly, the correlations between psychological well-being and health status [*r* (367) = 0.48, *p* < 0.001], as well as between psychological well-being and quality of life [*r* (372) = 0.65, *p* < 0.001], are significant. Therefore, higher scores for general health and quality of life are associated with higher scores for psychological well-being. The PCA and EFA analyses also confirmed, as previously discussed in the methodological section, that the items used for psychological well-being (measured by the WHO-5), perceived general health and quality of life measure a same underlying dimension, that we called ‘total well-being’.

#### 3.2.3 Perceived social support

As for the COPE index quality of support subscale, data analysis reveals that caregivers in our sample assigned an average score of 2 to 3 (corresponding to responses ranging from ‘sometimes’ to ‘often’) to items 1, 2, and 4. For item 2, the average rating fell between 3 (‘often’) and 4 (‘always’). This suggests that the caregivers in our sample reported feeling supported by friends, health and social services, and generally in their role sometimes or often; additionally, they indicated that they felt supported by their own family ‘often’ or ‘always’ (see Table 8). However, Table 8 also highlights that a significant percentage of respondents felt poorly supported by friends and neighbors (35.3% ‘never’ and 29.1% only ‘sometimes’), and by social and health services (26.4% ‘never’ and 42.7% only ‘sometimes’).

**Table 8:** Descriptive statistics and response frequencies for COPE index subscale measuring social support.

COPE index subscale for social support	*N*	Mean	Lower 95%CI	Upper 95%CI	SD	Variance	Never (1)		Sometimes (2)		Often (3)		Always (4)	
							*N*	%	*N*	%	*N*	%	*N*	%
1. Do you feel well supported by friends or neighbors?	382	2.13	2.03	2.24	1.043	1.087	135	**35.3**	111	**29.1**	86	22.5	50	13.1
2. Do you feel supported by your family?	381	3.32	3.24	3.41	0.860	0.740	17	4.5	48	12.6	111	**29.1**	205	**53.8**
3. Do you feel well supported by health and social services?	382	2.13	2.04	2.22	0.904	0.817	101	**26.4**	163	**42.7**	85	22.3	33	8.6
4. Overall, do you feel well supported in your role of caregiver?	386	2.77	2.68	2.86	0.871	0.758	28	7.3	116	**30.1**	158	**40.9**	84	21.8

Regarding perceived support, the t-test revealed no significant difference according to gender [*t* (369) = −0.57, *p* = 0.563]. Similarly, the ANOVA test applied to age did not show any statistically significant difference [*F* (4, 118) = 1.72, *p* = 0.149] (See Table 9).

**Table 9:** COPE Index subscale for social support: frequencies, percentages and statistical tests.

COPE index subscale for social support	*N*	Mean	Lower 95% CI	Upper 95% CI	SD	Statistical test	Statistical value
Gender						*T*-test	*t* = −0.57, df = 369, *p* = 0.563
Male	133	2.57	2.48	2.66	0.531		
Female	238	2.61	2.52	2.69	0.654		
Tot.	371	2.59	2.53	2.65	0.612		
Age						ANOVA	(*F* = 1.72, df1 = 4, df2 = 118), *p* = 0.149
70–79	57	2.78	2.61	2.94	0.626		
60–69	138	2.54	2.43	2.64	0.613		
50–59	102	2.60	2.49	2.70	0.546		
≥ 80	40	2.49	2.28	2.71	0.661		
Under 50	34	2.62	2.38	2.86	0.680		

*Social support* is a very multifaceted construct that encompasses both the overall perception of being supported by friends and family networks, as well as by health and social services. In the questionnaire administered to our sample, in addition to the COPE social support subscale, two other questions were included to assess whether caregivers could rely on someone in times of need. When asked if someone would be available to assist them in caring for the older adult person if they were ill, the majority of caregivers (44.8% + 43.8% = 88.6%) responded positively. Similarly, a significant proportion of caregivers (42.6% + 44.2% = 86.8%) also answered affirmatively when asked if there would be someone available to take care of the older adult person in their absence, allowing them a break from caregiving duties (see Table 10).

**Table 10:** Capacity to find a substitute.

	Yes, I could find someone quite easily		Yes, I could find someone, but with some difficulty		No, nobody	
Items	*N*.	%	*N*.	%	*N*.	%
If you were ill, would there be someone who could help you in caring for the older adult person?	173	44.8%	169	43.8%	44	11.4%
If you needed a break from caring, would there be someone who could take care of the older adult person in your place?	164	42.6%	170	44.2%	51	13.2%

No statistical differences were found regarding gender and age for both questions. As for gender, the *χ*^2^ for the first and second questions were respectively: *χ*^2^ (2, N = 386) = 1.20, *p* = 0.550, and *χ*^2^ (2, *N* = 385) = 1.85, *p* = 0.371; as for age, the *χ*^2^ for the first and the second questions were respectively: *χ*^2^ (8, *N* = 386) = 6.25, *p* = 0.619, and *χ*^2^ (8, *N* = 385) = 6.95, *p* = 0.542. In other words, caregivers’ personal belief that they can count on the support of someone else in case of illness or need for a break does not seem to be influenced by age or gender.

After presenting the data for each of the variables and dimensions studied, the following analyses are carried out to answer specifically and in more detail the three research questions underlying this paper.

#### 3.2.4 Caregiving burden and psychological well-being

Before addressing RQ1 (*Does caregiver burden affect psychological well-being? If so, to what extent?*), a correlation analysis was performed on the various measures used to evaluate caregiving burden and well-being. The results revealed significant associations, indicating that as caregiving burden increases, well-being decreases, and vice versa (see Table 11).

**Table 11:** Correlation matrix.

		4 items specifically designed for measuring care burden	COPE Index subscale for care burden	General health	Quality of life	Psychological well-being (WHO-5)
4 items specifically designed for measuring care burden	Pearson’s r	—				
	df	—				
	*p*- value	—				
COPE Index subscale for care burden	Pearson’s r	0.691***	—			
	df	375	—			
	*p*- value	< 0.001	—			
General health	Pearson’s r	−0.269***	−0.339***	—		
	df	378	370	—		
	*p*- value	< 0.001	< 0.001	—		
Quality of life	Pearson’s r	−0.457***	−0.546***	0.398***	—	
	df	381	372	376	—	
	*p*- value	< 0.001	< 0.001	< 0.001	—	
Psychological well-being (WHO-5)	Pearson’s r	−0.444***	−0.540***	0.484***	0.657***	—
	df	372	364	367	372	—
	*p*- value	< 0.001	< 0.001	< 0.001	< 0.001	—

*p < 0.05; **p < 0.01; ***; *p* < 0.001. Caregiving burden and well-being.

Specifically:

- Each of the three variables used to measure well-being is negatively and significantly correlated with the two variables measuring care burden.
- The two variables measuring care burden are – as already mentioned at the end of section 3.2.1 – positively correlated with each other, thus indicating that they capture different aspects of the same underlying dimension.
- The three variables measuring individual well-being - as already mentioned at the end of section 3.2.2 – are positively correlated with each other, demonstrating that they also capture different aspects of the same underlying dimension.

Given the highly significant results of internal reliability (Cronbach’s index), PA and EFA for both ‘total well-being’ (items of WHO-5, general health, and quality of life), and ‘total care burden’ (items specifically designed for testing care burden, and COPE subscale for caregiver burden), as pointed out in the methodological session, we also calculated the correlation between these two new variables. The analysis once again revealed a significant negative correlation (Pearson’s r = −0.582, *p* < 0.001) indicating that as care burden increases, well-being decreases. Moreover, linear regression analysis confirmed that changes in caregiver burden predict changes in caregiver well-being (*R*^2^ = 0.339; *F* = 184, *p* < 0.001).

Specifically addressing RQ1, we tested whether caregiver burden is a predictor of poor psychological well-being and at what point it leads to psychological distress, by conducting linear regression and receiver operating characteristic (ROC) analyses.

Linear regression analysis revealed that care burden is a significant predictor of psychological well-being, both when the four items specifically designed are taken into account (*R*^2^ = 0.197; *F* = 91.3, *p* < 0.001) and when the Cope index subscale is used (*R*^2^ = 0.290; *F* = 150, *p* < 0.001). These results indicate that as caregiving burden increases, psychological well-being significantly decreases.

To identify the point at which perceived fatigue leads to a decline in psychological well-being, we also conducted a ROC analysis, assuming a value ≤13 on the WHO-5 scale as the cut-off for poor psychological well-being. We sought to determine at what average level of caregiver burden (as measured separately by the COPE subscale and by the four items specifically designed) our sample began to exhibit poor psychological well-being. Operationally, we considered the WHO-5 scale as a dichotomous dependent variable (scores above 13 indicate good well-being, scores equal to or below 13 indicate poor well-being) and as covariates once for the COPE caregiver burden subscale and once for the four items specifically designed for measuring care burden.

**Figure 1:**
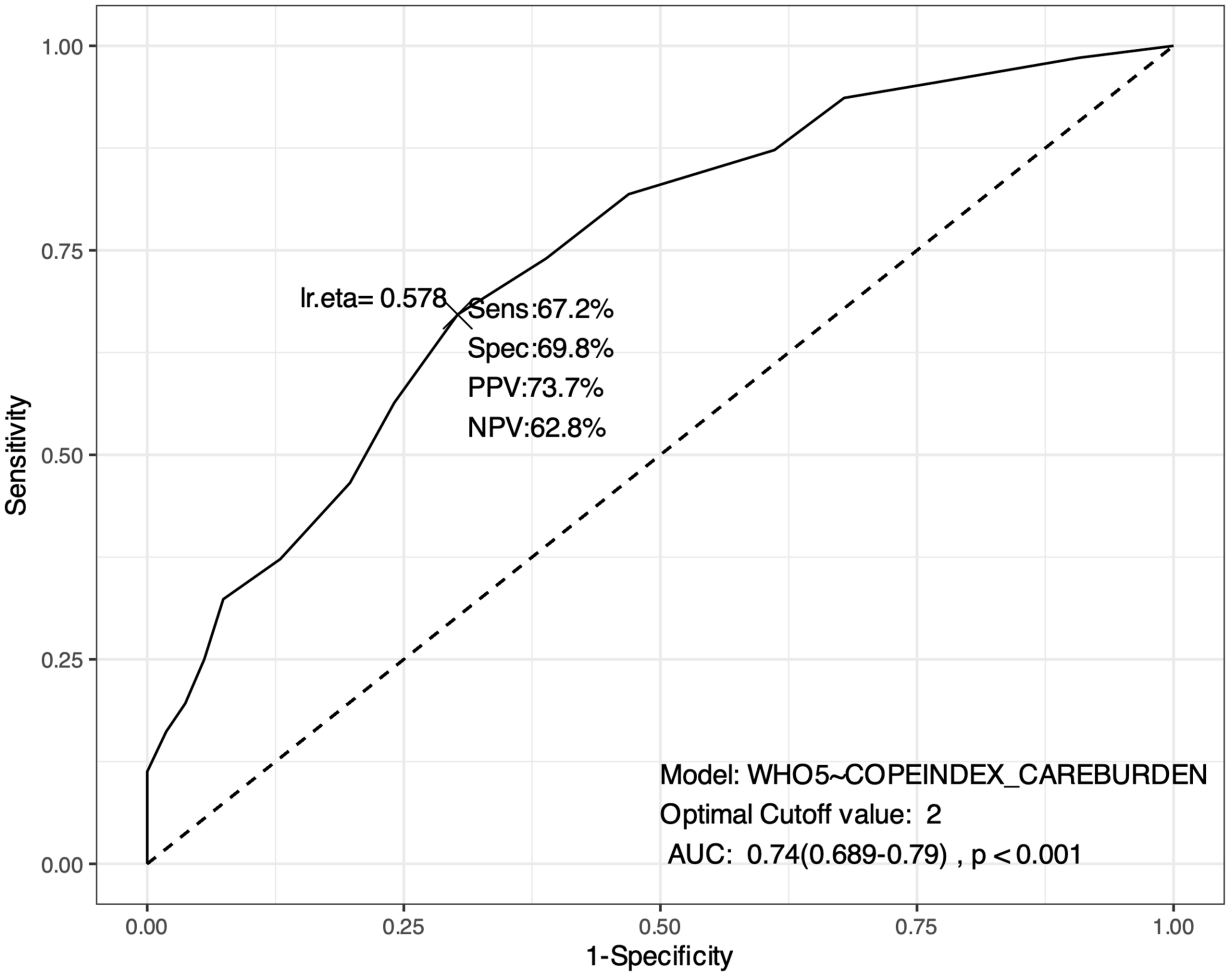
ROC Curve. Identification of cut-off value for caregiving burden (measured by COPE subscale). *This ROC analysis suggests that the model has a reasonably good performance in discriminating between different levels of psychological well-being based on caregiving burden, with a good balance between sensitivity (the model’s ability to identify true positives, i.e., cases where the psychological well-being is correctly identified given the care burden) and specificity (the model’s ability to identify true negatives, i.e., cases where psychological well-being is correctly identified as unaffected by caregiving burden). The AUC: 0.74 (0.689–0.79), *p* < 0.001 indicates a good level of accuracy of the model in discriminating between those who have low psychological well-being and those who do not.

As for the COPE subscale for care burden, the ROC analysis identified an optimal cut-off value of 2 (on a 4-point Likert scale ranging from 1 to 4, with 1 = ‘never’; 2 = ‘sometimes’; 3 = ‘often’; 4 = ‘always’). This means that a score above 2 (frequency ‘sometimes’ or more) indicates a higher probability of low psychological well-being, while a score below 2 may correspond to a lower probability of low psychological well-being. In other words, a value of 2 is the point at which the burden of care (measured by the COPE subscale) begins to predict a significant reduction in psychological well-being (see Figure 1).

**Figure 2:**
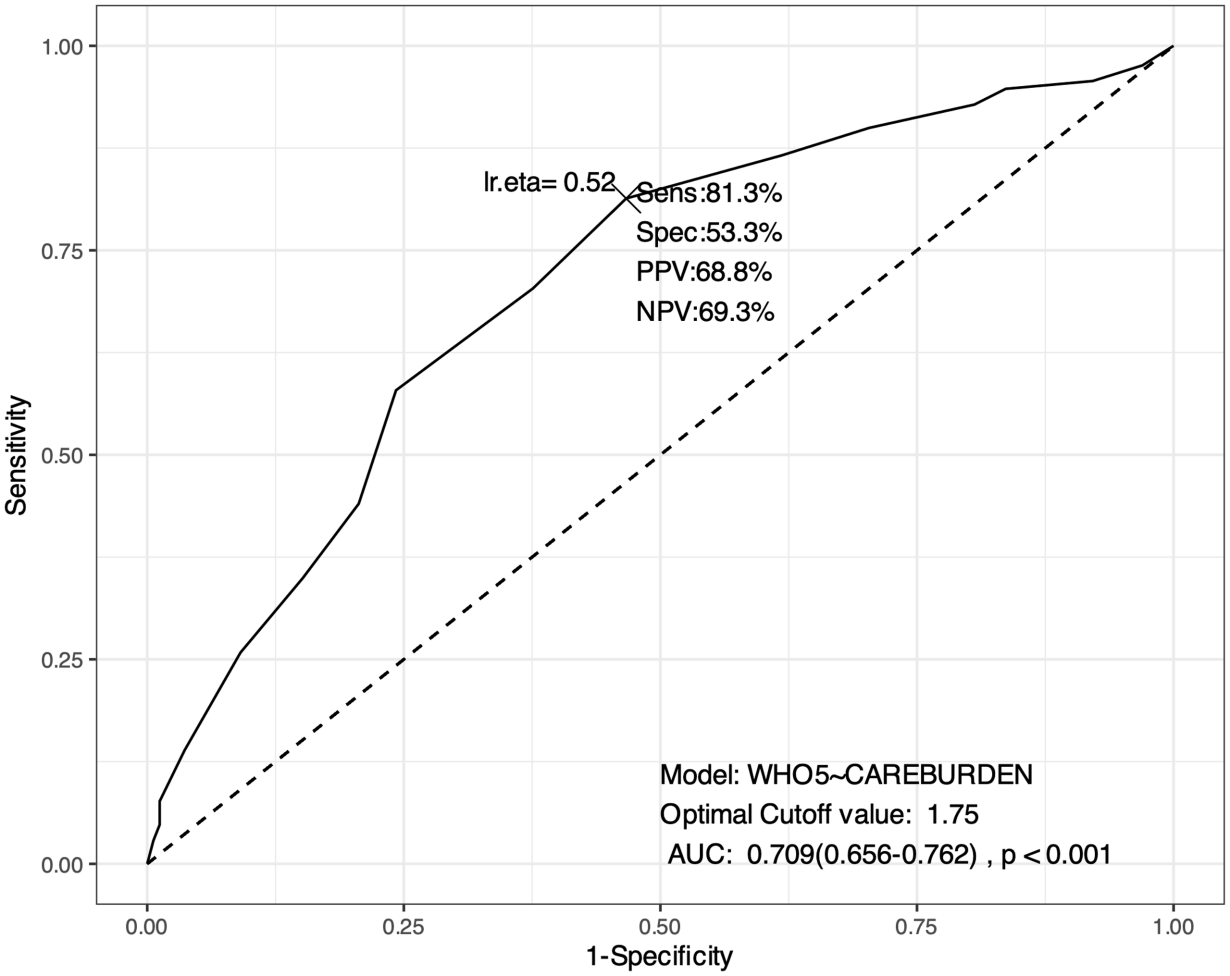
ROC Curve. Identification of cut-off value for caregiving burden (measured by the four specifically designed items). *This ROC analysis suggests that the model has a reasonably good performance in discriminating between different levels of psychological well-being based on caregiving burden, with a good balance between sensitivity (the model’s ability to identify true positives, i.e., cases where the psychological well-being is correctly identified given the care burden) and specificity (the model’s ability to identify true negatives, i.e., cases where psychological well-being is correctly identified as unaffected by caregiving burden). The AUC: 0.709 (0.656–0.762), p < 0.001 indicates a good level of accuracy of the model in discriminating between those who have low psychological well-being and those who do not.

As for the mean value of the four items specifically designed for measuring care burden, the ROC analysis identified an optimal cut-off the value of 1.75 (on a 5-point Likert scale ranging from 0 to 4, with 0 = ‘never’; 1 = ‘rarely’; 2 = ‘sometimes’; 3 = ‘often’; 4 = ‘almost always’). In this case, a score above 1.75 signals a higher probability of low psychological well-being, while a score below 1.75 signals a lower probability of low psychological well-being. In other words, a value of 1.75 is the point at which the burden of care begins to predict a significant reduction in psychological well-being (see Figure 2).

These results can be used to identify individuals who may need additional support to improve their psychological well-being when experiencing a significant caregiving burden (equals 2 when measured with the COPE subscale and 1.75 when measured with the four specifically developed items).

#### 3.2.5 Social support and psychological well-being

To address the first part of RQ2 (*Does social support influence psychological well-being?*), correlation and regression analyses were carried out. As expected, correlation analysis suggests that perceived social support (measured by the COPE subscale index) positively impacts carers’ psychological well-being (measured by WHO-5) [*r* (357) = 0.348, *p* < 0.001] (Table 12).

**Table 12:** Correlation matrix.

		Psychological well-being (WHO-5)	COPE Index subscale for social support
Psychological well-being (WHO-5)	Pearson’s r	—	
	df	—	
	*p*- value	—	
COPE Index subscale for social support	Pearson’s r	0.348***	—
	df	357	—
	*p*- value	< 0.001	—

*p < 0.05; **p < 0.01; ***p < 0.001. Social support and psychological well-being.

Linear regression analysis supports this, indicating that social support is a significant predictor of psychological well-being (*R*^2^ = 0.121; *F* = 49.2, *p* < 0.001).

#### 3.2.6 Multivariate logistic regression

Multivariate logistic regression was conducted to further explore the relationship between perceived caregiver burden and psychological well-being, specifically identifying the weight of each aspect of caregiving on psychological well-being, as well as to identify which dimensions of social support have the greatest impact on perceived psychological well-being, thus addressing the second part of RQ2 (*which aspects of social support have the most significant impact on caregivers’ psychological well-being?*).

In the table below, the ‘Estimate’ column shows the coefficients in log-odds form. More precisely, the estimation output highlights whether the effect of the predictors on the variable of interest is positive or negative.

Table 13 shows only those items from the different scales that have a significant impact on the dependent variable (i.e., psychological well-being exclusively measured with WHO-5).

**Table 13:** Aspects of social support having the greatest impact on psychological well-being.

Multivariate logistic regression						
		Value	Estimate	SE	*z*- value	Pr(>|z|)
4 items specifically designed for measuring care burden	Feeling stressed about caring and coping with other responsibilities	0	1.9431	0.6129	3.171	0.00152**
	Feeling stressed about caring and coping with other responsibilities	1	1.4118	0.5526	2.555	0.01062**
	Feeling stressed about caring and coping with other responsibilities	2	0.8232	0.4976	1.654	0.09806*
	Feeling fatigued in caring	0	1.47032	0.64318	2.286	0.0223**
	Feeling fatigued in caring	1	1.01750	0.54053	1.882	0.0598*
	Feeling insecure in caring	0	1.2818	0.6516	1.967	0.04916*
COPE index subscale (care burden)	Assisting causes difficulties with friends	4	1.5382	0.6292	2.445	0.0000***
	Assisting negatively impacts on the state of physical health	4	0.6495	0.4826	1.346	0.0885*
	Assisting causes difficulties in relations with your family	4	0.4836	0.0863	5.604	0.0000***
	Assisting cause economic difficulties	4	**−0.5927**	0.0636	−9.319	0.0000***
COPE index subscale (social support)	Feeling supported by health and social services	1	0.5923	0.2764	2.1429	0.0162**
	Feeling supported by health and social services	2	0.6238	0.3097	2.0142	

Regarding the four items specifically designed for measuring care burden, Table 13 shows that:

- Lower levels of stress in caring and coping with responsibilities (‘Do you feel stressed between caring for the older adult person and trying to cope with other responsibilities?’ with 0 = ‘never’, 1 = ‘rarely’, and 2 = ‘sometimes’) have a significant impact on psychological well-being; hence, less stress corresponds to greater well-being. Although the relationship is significant for all values of the variable, it is observed that the significance decreases when the variable takes values of 1 and 2. In other words, at values of 1 and 2, the variable loses its significance, as a higher level of stress would have a negative effect on well-being.
- Lower levels of fatigue in caring (‘Do you feel fatigued when caring for the older adult? with 0 = ‘never’, and 1 = ‘rarely’) also have a significant effect on psychological well-being; in other words, lower levels of perceived fatigue correspond to higher psychological well-being.
- Lower levels of insecurity in caring (‘Do you feel insecure about what to do for your older adult person?’ with 0 = ‘never’) also correspond to greater psychological well-being.

Additionally, regarding caregiving burden, as measured by the COPE index subscale, the reported absence of perceived difficulties in relationships with friends (*‘Does caring cause any difficulties in your relationships with friends?’*) and family (*‘Does caring cause any difficulties in your relationships with family?’*), as a result of caregiving tasks, and the absence of a negative effect of caring on personal physical health (*‘Does caring have a negative effect on your physical health status?’*) also have a significant impact on psychological well-being. In other words, the less people feel that caring has a negative impact on their relationships (with friends and family) and on their own physical health, the better their self-reported psychological well-being. Conversely, greater financial difficulties (*‘Does providing assistance cause you financial difficulties?’*) have a negative impact on psychological well-being.

On the COPE Index social support subscale, only the item concerning social and health services (*Do you feel adequately supported by health and social services (public, private, or voluntary?)*) is significantly associated with well-being at higher levels. In other words, the greater the perceived support from social and health services, the greater the reported psychological well-being. This information could be used to enhance social and health services, given their crucial role in the psychological well-being of caregivers. If psychological well-being is closely related to the extent to which caregivers feel supported by these services, then improving the quality of services should increase the likelihood of caregivers achieving good levels of psychological well-being.

#### 3.2.7 Mediation analysis

After addressing the first two RQs, a mediation analysis was conducted using the following variables:

- WHO-5 (dependent variable);
- Subscale COPE index for care burden (predictor);
- Subscale COPE index for social support (mediator).

**Figure 3:**
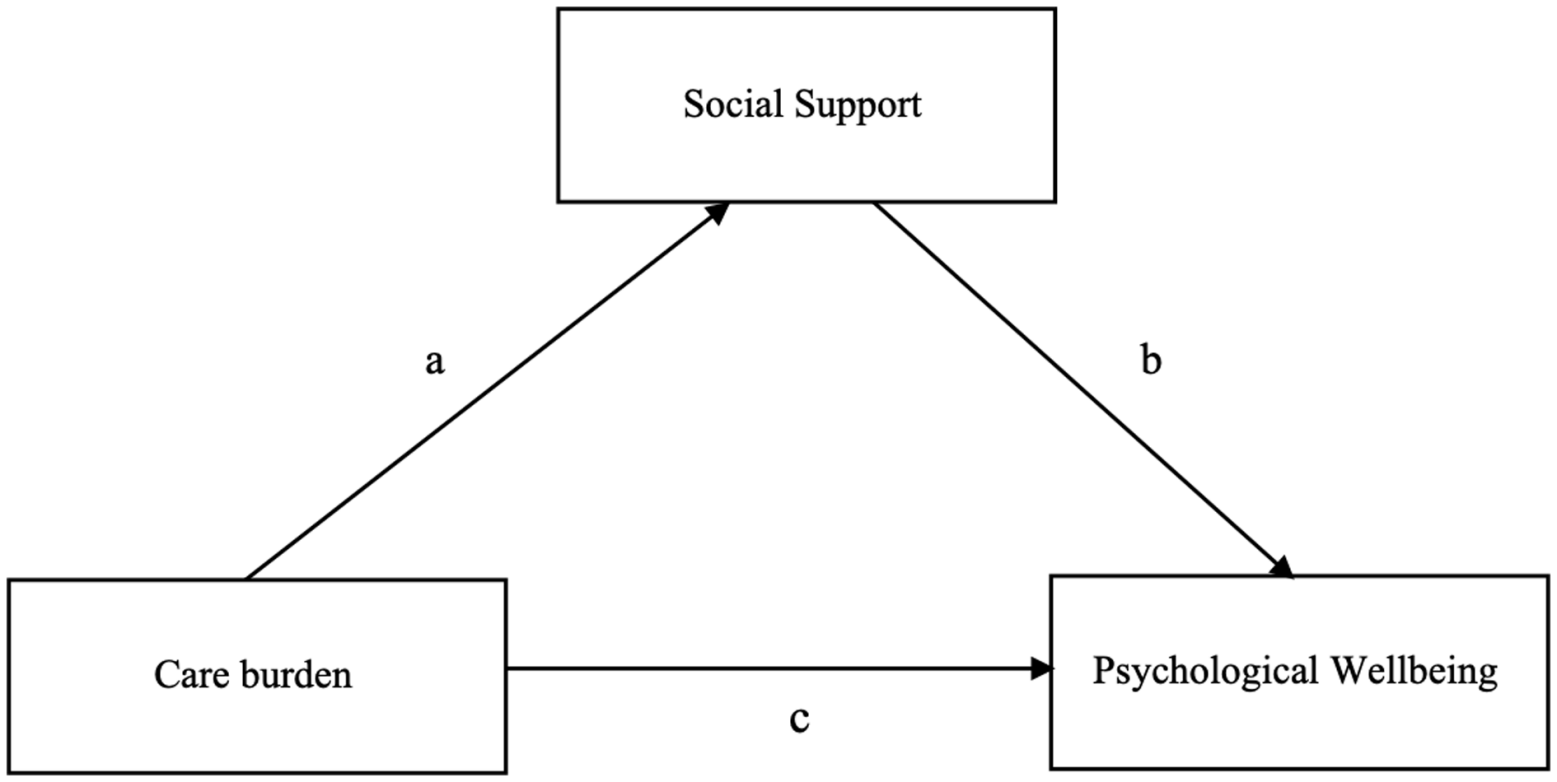
Mediation model.

This analysis aimed to answer RQ3 (*If caregivers’ burden negatively affects their psychological well-being, does the perception of social support mediate this relationship?*) (Figure 3).

The analysis revealed a significant direct and indirect effect of the care burden on psychological well-being, with the direct effect accounting for the majority of the total effect (88%). Although social support plays a smaller and partial role in explaining the relationship between care burden and psychological well-being (12%), it is nonetheless statistically significant (*p* = 0.001; Table 14). Therefore, although social support does not neutralize the negative impact of caregiver burden on psychological well-being, it contributes to buffering (i.e., reducing) its negative impact, confirming the importance of social support as a mitigating factor. The estimates patch confirms that: the greater the care burden experienced, the lower the perceived social support (a); the greater the social support experienced, the greater the psychological well-being (b); the greater the care burden experienced, the lower the psychological well-being experienced (c).

**Table 14:** Mediation estimates and path estimates.

		Label	Estimate	SE	*Z*	*p*	% Mediation
Mediation estimates	Effect						
	Indirect	a × b	−0.0897	0.0273	−3.29	0.001	12.0
	Direct	c	−0.6560	0.0649	−10.11	< 0.001	88.0
	Total	c + a × b	−0.7457	0.0611	−12.20	< 0.001	100.0
Path estimates	Path						
	Subscale COPE care burden → Subscale COPE social support	a	−0.524	0.0675	−7.77	< 0.001	
	Subscale COPE social support → Psychological well-being (WHO-5)	b	0.171	0.0472	3.63	< 0.001	
	Subscale COPE care burden → Psychological well-being (WHO-5)	c	−0.656	0.0649	−10.11	< 0.001	

The buffering role of social support (measured through the COPE Index subscale) in mediating the relation between care burden (measured through the COPE Index subscale) and psychological well-being (measured via WHO-5).

Even if we calculate the mediation analysis using the four items specifically designed to measure care burden as a predictor, the results would be similar (Table 15). In this case, although the direct effect accounts for the majority of the total effect (83.5%), social support plays a slightly larger, though still partial, role in explaining the relationship between care burden and psychological well-being (16.5%). As the *p*-value (0.001) indicates, this role is statistically significant.

**Table 15:** Mediation estimates and path estimates.

		Label	Estimate	SE	*Z*	*p*	% Mediation
Mediation estimates	Effect						
	Indirect	a × b	−0.0943	0.0252	−3.74	< 0.001	16.5
	Direct	c	−0.4768	0.0612	−7.79	< 0.001	83.5
	Total	c + a × b	−0.5712	0.0589	−9.70	< 0.001	100.0
Path estimates	Path						
	Care burden measured by the four questions specifically designed → Subscale COPE social support	a	−0.441	0.0622	−7.09	< 0.001	
	Subscale COPE social support → Psychological well-being (WHO-5)	b	0.214	0.0487	4.39	< 0.001	
	Care burden measured by the four questions specifically designed → Psychological well-being (WHO-5)	c	−0.477	0.0612	−7.79	< 0.001	

The buffering role of social support (measured through the COPE Index subscale) in mediating the relation between care burden (measured through the four questions specifically designed) and psychological well-being (measured via WHO-5).

## 4 Discussion

Family caregivers play a crucial role in caring for frail older people. Research shows that caregiving can have both positive and negative effects on caregivers’ well-being. On the one hand, providing care may enhance the caregivers’ sense of purpose and satisfaction, particularly when the role is taken voluntary. Studies, such as those by Weinstein and Ryan (46), and by Hui et al. (47), suggest that pro-social behavior can improve well-being for both caregivers and recipients. Other research confirms that caring for older relatives can foster feelings of fulfillment and satisfaction [e.g., (12–15, 17)]. However, when the demands of caregiving and the related burden exceed individual resources, it can lead to negative outcomes. Thus, caregivers, particularly family caregivers of older adults, often face significant physical, emotional, and psychological strain. This is well-documented in studies showing a decline in caregivers’ quality of life and psychological well-being [e.g., (2, 5–7, 9, 54)]. In this complex situation, the perception of social support – whether from other family members, friends, or services – can play a crucial role in reducing stress [e.g., (25)] and improving the psychological well-being of family caregivers of older adults [e.g., (23, 24)].

RQs that guided this study aimed to understand whether caregiving burden negatively impacts individual psychological well-being and, conversely, whether social support has a positive influence, while also exploring these relationships more thoroughly.

## 5 Conclusion

### 5.1 Caregiving burden

Regarding *caregiver burden,* a significant portion of our sample reported high levels of time deprivation, fatigue, feelings of imprisonment, and difficulties in maintaining social relationships. These findings are consistent with previous research, such as that by Rokicka, and Zajkowska (48), who found that carers of older adults, especially co-residents, often sacrifice personal activities and social interaction. An unbalanced trade-off between time devoted to caring for others and time devoted to oneself can be detrimental to health, life satisfaction and well-being. Returning to our research, although our analysis did not reveal any significant gender differences, women generally reported higher levels of stress and more pronounced declines in their physical and mental health compared to men. Similarly, older caregivers also reported higher levels of perceived fatigue, although advancing age *per se* did not emerge as a reliable predictor of perceived fatigue.

### 5.2 Psychological well-being

In terms of psychological *well-being*, as measured by the WHO-5, our findings are consistent with those of Santini et al. (49) in a similar population (i.e., 100 family caregivers of older adult people with incontinence), but lower than the average score measured in an Italian sample from the general population by Carrozzino et al. (32), which reported an average score of 11.64 ± 4.95. In our study, 55.9% of caregivers also scored below the reference cut-off of 13, indicating poor psychological well-being. This highlights the pervasive issue of low psychological well-being among family caregivers in similar caregiving roles. No significant gender differences were found. However, age emerged as a significant predictor of well-being, suggesting that the advancing age of caregivers predicts a significant decline in their psychological well-being, as well as in their perceived health and quality of life.

### 5.3 Social support

In line with the results of other research on family caregivers of older people [e.g., (25)], a significant proportion of our respondents reported feeling poorly supported by friends, neighbors, and social and health services, irrespective of gender and age. Despite this, only a small percentage of caregivers reported that they could not find a replacement in case of illness or need for a break. Conversely, many respondents mentioned having someone available to take over caregiving duties in their absence, allowing them some respite.

### 5.4 Caregiving burden and psychological well-being

Addressing RQ1, our analysis aligns with the findings of a systematic review by Del-Pino-Casado et al. (50), who identified caregiver burden as a key risk factor for depressive symptoms in caregivers of older adults. Our findings revealed a significant correlation between increased caregiving burden and decreased psychological well-being. Focusing solely on the results from the analysis of the responses provided by our sample to the validated scales items (COPE Index subscale for care burden and WHO-5 for psychological well-being), a significant negative correlation was found between caregiving burden and psychological well-being [*r* (364) = −0.540, *p* < 0.001], with caregiving burden emerging as a significant predictor of reduced psychological well-being (*R*^2^ = 0.290; *F* = 150, *p* < 0.001). Through ROC analysis, we determined a threshold value (which for the COPE Index subscale is equal to 2 on a 1–4 scale) at which caregiver burden begins to negatively impact psychological well-being. As previously mentioned, the results confirmed that caregiver burden does not significantly impact caregivers’ psychological well-being as long as it does not exceed a certain level. These findings have critical implications, suggesting that interventions to support caregivers facing significant burdens could prevent deterioration or worsening of their psychological well-being. This result also suggests that regular monitoring of caregiving burden and early intervention with psychosocial support could help prevent caregivers from reaching a critical point that could lead to more severe psychological outcomes. Moreover, in our sample, multivariate logistic regression allowed us to identify the specific factors of caregiving burden that seem to most significantly impact psychological well-being. The analysis revealed that psychological well-being is higher when caregivers experience lower levels of stress, fatigue, and insecurity in their caregiving roles, and when caregiving tasks have a reduced impact on their relationships. These findings confirm the need to monitor caregiving burden in specific personal and relational domains of caregivers of frail older people with LTC needs. The aim should be to keep levels of stress, fatigue and personal insecurity low, to limit the impact of caregiving burden on relationships, and to intervene promptly when these levels are exceeded.

### 5.5 Social support and psychological well-being

As for RQ2, the relationship between perceived social support and psychological well-being was also found to be highly significant: greater perceived social support correlates with better psychological well-being. Focusing strictly on the results of the analysis of responses provided by our sample to the validated scale items (COPE Index subscale for social support and WHO-5 for psychological well-being), a positive correlation was found between greater perceived social support and better psychological well-being [*r* (357) = 0.348, *p* < 0.001], with social support emerging as a significant predictor of psychological well-being (*R*^2^ = 0.121; *F* = 49.2, *p* < 0.001).These results are partially consistent, among others, with the findings of Leung et al. (51), who demonstrated that support from friends significantly reduces caregiver burden and improves mental health outcomes. Similarly, Muñoz-Bermejo et al. (58) highlighted that the perceived social support can contribute to improving mental well-being, especially for older caregivers. De Maria et al. (52) also observed that perceived support from family and friends improved the health-related quality of life of both older adults and their informal caregivers. In our sample, multivariate logistic regression analysis emphasized that support from health and social services plays a critical role in determining caregivers’ psychological well-being. While support from friends, family and neighbors is important, formal services emerged as the most significant factor. The greater the perception of support from services, the higher the reported psychological well-being.

### 5.6 The mediating role of social support between care burden and psychological well-being

Finally, concerning RQ3, mediation analysis confirmed that social support plays a buffering role in mitigating the negative impact of caregiving burden on psychological well-being. Even though caregiving can be demanding, knowing that one can rely on social support – mainly from services – helps in reducing its adverse effects on caregivers’ psychological well-being.

Overall, these findings underline the importance of robust social support networks and, in particular, the urgency of investing in health and social services to alleviate the psychological burden of caregivers and improve their general well-being. In other words, increased public investment in health and social services could enhance the well-being of caregivers and, consequently, that of the older adults they care for.

### 5.7 Limitations and future research

While this study provides valuable insights, several limitations should be acknowledged.

First, the generalizability of the findings is limited due to the sample of caregivers, which is not representative of the Italian population of family caregivers of frail older adults. Furthermore, data collection was conducted in a single session, preventing the calculation of test–retest reliability, which limits the robustness of the results. In addition, although the items of the scales used (WHO-5 and the two subscales of the COPE index) have been shown to adequately measure the latent factors (psychological well-being, caregiver burden and social support), they do not always seem to fully fit our data. This may be due to several factors, including the limited number of items on each scale, as well as, the socio-demographic differences of our sample, the differing contexts in which the surveys were administered and completed (either at home or in a union office), which may have influenced the atmosphere and comfort levels of the participants. Another limitation relates to the format of the questions (all closed), which may have limited the carers’ ability to fully express their attitudes, perceptions, and viewpoints. Moreover, although guidelines recommend administering a depression screening when low psychological well-being is detected (as was the case for many in our sample), this step was not implemented, highlighting a significant gap between research knowledge and practical intervention.

Future research should aim to overcome these limitations by adopting more robust methodologies, but it should also aim to achieve new goals. A longitudinal approach, for instance, could involve measuring key variables before and after specific training or support interventions. Additionally, comparative studies could be useful, such as comparing informal caregivers of older adult individuals with formal caregivers or contrasting caregivers of older adult people with those caring for individuals with different needs (e.g., children with special needs). Cross-cultural studies would also be insightful, allowing for comparisons of psychological well-being among caregivers in countries with different healthcare and social support systems.

### 5.8 Implications for policy

A shift in the paradigm for managing interventions to support families dealing with the challenges of frailty and LTC seems urgent. This change should embrace a community-based approach to reduce the isolation in which carers often find themselves, as caring responsibilities are often seen as an ‘essentially private affair’ within Italian society. This change would alleviate some of the burden traditionally placed on family members.

The findings of this study offer valuable practical insights. Firstly, it highlights the importance of maintaining ongoing communication between caregivers and service providers. Listening to caregivers’ needs and regularly monitoring their psychological health are essential to promptly address potential risks. Neglecting such risks can adversely affect both caregivers and those they care for. Secondly, the study underscores the need to invest in developing and offering training programs for family caregivers. These programs should equip them with practical skills to effectively manage the needs of older assisted relatives and cope with the stress of their duties, thus reducing both the perceived burden and the likelihood of adverse psychological outcomes. This becomes even more urgent in contexts like ours, where a high percentage of caregivers are older adult themselves or approaching this stage of life. Improving access to services and training programs, including overcoming barriers related to service locations, is critical to ensuring that caregivers can fully utilize available resources.

## Data Availability

The data analyzed in this study is subject to the following licenses/restrictions: The data presented in this study are available upon request from the corresponding author. The data are not publicly available because they are still the object of ongoing analyses. Requests to access these datasets should be directed to Ramona Bongelli, ramona.bongelli@unimc.it.

## Footnotes

^1^https://www.istat.it/wp-content/uploads/2022/09/REPORT-PREVISIONI-DEMOGRAFICHE-2021.pdf, last accessed 18 September 2024.

^2^Available at https://dictionary.apa.org/frail-elderly, last accessed 25 September 2024.

^3^WHO definition for long-term care, available at https://www.who.int/europe/news-room/questions-and-answers/item/long-term-care, last accessed 26 September 2024.

^4^https://www.psykiatri-regionh.dk/who-5/Documents/WHO5_Italian.pdf; last accessed on 24 March 2024.

^5^Retrieved from https://www.jamovi.org

^6^Retrieved from https://cran.r-project.org
