# Supplementary material for: Caregiving burden, social support, and psychological well-being among family caregivers of older Italians: a cross-sectional study
Source: Front Public Health. 2024 Oct 23;12:1474967. doi: 10.3389/fpubh.2024.1474967 (PMC11537916; doi:10.3389/fpubh.2024.1474967)
Supplement: Supplementary file 1 [file Table_1.docx]

Supplementary Table

Table 1: Sociodemographic characteristics of family caregivers

|  | **N** | **%** | **Mean** |
| --- | --- | --- | --- |
| ***Gender*** |  |  |  |
| Female | 251 | 64.9% |  |
| Male | 136 | 35.1% |  |
| Tot. | 387 | 100% |  |
| ***Age (63.3; SD 11.4)*** |  |  |  |
| under 50 | 36 | 9.3% |  |
| 50-59 | 104 | 26.9% |  |
| 60-69 | 144 | 37.2% |  |
| 70-79 | 59 | 15.2% |  |
| ≥ 80 | 44 | 11.4% |  |
| Tot. | 387 | 100% |  |
| ***Level of education*** |  |  |  |
| no educational qualification | 6 | 1.6% |  |
| primary school | 59 | 15.2% |  |
| junior high school | 106 | 27.4% |  |
| senior high school | 167 | 43.2% |  |
| Post-secondary degree (non-tertiary) | 2 | 0.5% |  |
| Tertiary vocational degree | 4 | 1.0% |  |
| Bachelor degree | 7 | 1.8% |  |
| Master degree | 34 | 8.8% |  |
| PhD | 2 | 0.5% |  |
| Tot. | 387 | 100% |  |
| ***Marital status*** |  |  |  |
| Married / Domestic partner | 273 | 70.5% |  |
| Unmarried | 58 | 15.0% |  |
| Divorced/separated | 36 | 9.3% |  |
| Widower/widow | 20 | 5.2% |  |
| Tot. | 387 | 100% |  |
| ***Relative relationship to the assisted person*** |  |  |  |
| Spouse/Partner | 83 | 21.4 % |  |
| Daughter/son | 242 | 62.5 % |  |
| Sibling | 13 | 3.4 % |  |
| Daughter/son-in-law | 27 | 7.0 % |  |
| Nephew | 8 | 2.1 % |  |
| Cousin | 1 | 0.3 % |  |
| Brother/sister-in-law | 1 | 0.3 % |  |
| Grandchild | 11 | 2.8 % |  |
| Other relationship | 1 | 0.3 % |  |
| Tot. | 387 | 100% |  |
| ***Residence*** |  |  |  |
| In the same house | 215 | 55.6 % |  |
| In different flats but in the same building | 50 | 12.9 % |  |
| Within walking distance | 65 | 16.8 % |  |
| A 10-minute journey by car, bus or train | 44 | 11.4 % |  |
| A 30-minute journey by car, bus or train | 11 | 2.8 % |  |
| No more than 1 hour away by car, bus or train | 1 | 0.3 % |  |
| More than 1 hour away by car, bus or train | 1 | 0.3 % |  |
| Tot. | 387 | 100% |  |
| ***Other non-family caregivers*** |  |  |  |
| NO | 219 | 56.6% |  |
| Yes, NOT cohabiting | 66 | 17.1% |  |
| Yes, cohabiting | 88 | 22.7% |  |
| Both (cohabiting + non cohabiting) | 7 | 1.8% |  |
| Missing | 7 | 1.8% |  |
| Tot. | 387 | 100% |  |
| ***Hours per week dedicated to the assistance of the elderly person*** |  |  |  |
| Male | 132 | 34.8% | 61.7 |
| Female | 247 | 65.2% | 60.3 |
| Tot. | 379 | 100% | 60.8 |

**
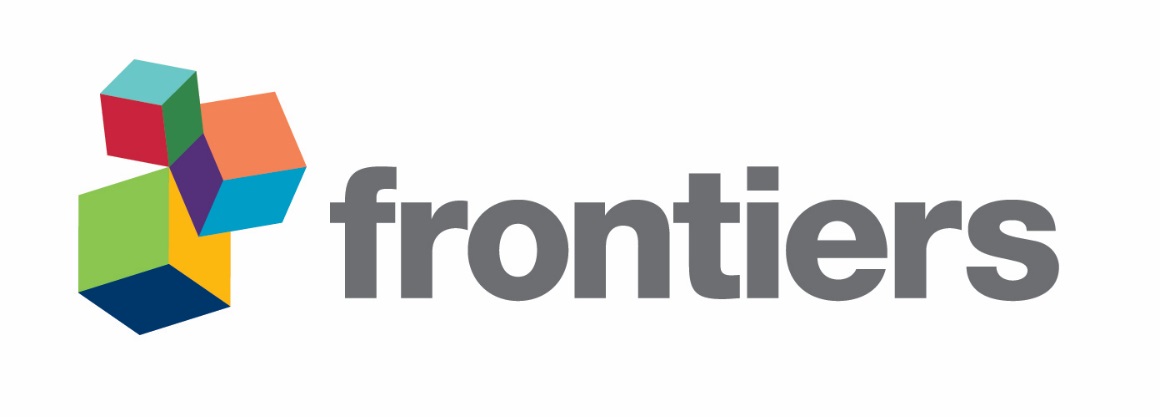
**
